# Clinical Performance of a Contemporary Hybrid Surface Implant System—A Prospective Pilot Study

**DOI:** 10.3390/jcm15218691

**Published:** 2026-11-07

**Authors:** Eswar Kandaswamy, Jeanne St Germain, Zixin Li, Qingzhao Yu, David Simmons, John J. Frazier

**Affiliations:** 1 Department of Periodontics, School of Dentistry, Louisiana State University Health Sciences Center, New Orleans, LA 70119, USA; jstge1@lsuhsc.edu (J.S.G.); dsimmo@lsuhsc.edu (D.S.); 2 Biostatistics and Data Science, School of Public Health, LSU Health-New Orleans, New Orleans, LA 70112, USA; zli5@lsuhsc.edu (Z.L.); qyu@lsuhsc.edu (Q.Y.); 3 Department of Radiology, School of Dentistry, Louisiana State University Health Sciences Center, New Orleans, LA 70119, USA; jfraz2@lsuhsc.edu

**Keywords:** dental implant, immediate loading, insertion torque, patient reported outcomes

## Abstract

**Background/Objectives**: Contemporary implant designs incorporating progressive macrogeometry and hybrid surface characteristics are intended to enhance primary stability while supporting favorable peri-implant tissue outcomes. This prospective cohort study evaluated the 2-year clinical, radiographic, prosthetic, and patient-reported outcomes of a contemporary hybrid surface tapered implant system with progressive macrogeometry. **Methods**: Thirty participants received 31 implants and were prospectively followed. Twenty-nine participants (30 implants) completed the 2-year follow-up evaluation. The study was prospectively registered at ClinicalTrials.gov (NCT05498662). Treatment protocols, including adjunctive grafting and loading, were selected according to predefined clinical criteria. Outcomes included implant survival and success, implant stability, peri-implant clinical parameters, marginal bone levels, prosthetic complications, and patient-reported satisfaction. Standardized radiographs were obtained longitudinally to assess marginal bone-level changes. **Results**: No implant failures were observed during the study period, and implant success was 96.7% at 2 years. Mean probing depths remained stable, measuring 2.91 ± 0.91 mm at 1 year and 2.86 ± 1.21 mm at 2 years. Marginal bone levels also remained stable between prosthesis delivery and the 1-year follow-up (mean change: 0.00; *p* > 0.05) and showed a small coronal shift between the 1- and 2-year evaluations (mean change: −0.17; *p* < 0.001). Prosthetic complications occurred in 16% of sites. Patient-reported satisfaction remained high throughout follow-up. **Conclusions**: Within the limitations of this small, single-center prospective cohort study, the evaluated implant system demonstrated acceptable clinical, radiographic, and patient-reported outcomes. Larger prospective studies are needed to confirm these findings.

## 1. Introduction

The global prevalence of tooth loss is a public health problem resulting in a compromise in an individual’s ability to function, smile, and masticate food [1,2,3]. Dental implant therapy is an important treatment option to rehabilitate missing teeth and restore form and function [4,5], and they exhibit a very high survival rate at over 90% [6,7]. An important consideration regarding prosthetic rehabilitation of dental implants is the timing of loading with protocols, which range from immediate to early and delayed loading [6,8]. During the last two decades, there has been considerable interest in the development of implant systems and loading protocols that permit immediate or early loading, with the aim of reducing the time frame between implant placement and prosthesis delivery [9,10,11]. The decision to implement immediate loading protocols is influenced by factors such as the functional status of the remaining dentition, the patient’s health, esthetic considerations, bone quality, the need for adjunctive hard or soft tissue grafting, as well as the primary stability of the implant [8,12].

Primary stability as measured by insertion torque and the measurement of implant stability quotient (ISQ) are two important drivers for immediate loading protocols [8,13,14]. Primary stability is governed by several site-level factors such as the quality and quantity of available bone but also by the macro- and microscopic features of a dental implant [15,16]. With recent technological advances in dental implant geometry, several new implant designs have been introduced that have deeper threads along the implant surface to permit higher insertion torque and improved implant stability quotient (ISQ) readings at placement. However, more recently concerns have arisen regarding the maintenance of these deeper exposed threads following crestal remodeling or the onset of peri-implantitis [17,18,19]. In this study, a contemporary tapered implant system (T3^®^ PRO implant system, ZimVie, Palm Beach Gardens, FL, USA) was evaluated. The implant incorporates a progressive thread design and a dual acid-etched surface; however, the purpose of the present study was not to evaluate the contribution of specific design features but rather to prospectively assess overall clinical, radiographic, prosthetic, and patient-reported outcomes associated with its use in routine clinical practice.

While numerous studies have investigated individual surgical, loading, and restorative protocols, fewer prospective studies have reported comprehensive clinical, radiographic, prosthetic, and patient-reported outcomes for a contemporary implant system used across clinician-selected treatment approaches. Such data are important because implant therapy is routinely performed under a variety of site conditions and treatment protocols that may not be fully represented in protocol-specific clinical studies. Accordingly, additional prospective evidence describing overall implant performance in routine clinical practice remains warranted.

Hence, the aim of this prospective cohort study was to evaluate the 2-year clinical, radiographic, prosthetic, and patient-reported outcomes of a contemporary hybrid surface implant system. The primary outcome was radiographic marginal bone-level change from prosthesis delivery through 1 and 2 years of follow-up, while secondary outcomes included implant survival and success, implant stability, peri-implant clinical parameters, prosthetic complications, and patient-reported satisfaction.

## 2. Materials and Methods

The protocol for this study was approved by the LSUHSC IRB (IRB #4572). The study was conducted in accordance with the Declaration of Helsinki and in compliance with the International Conference on Harmonization–Good Clinical Practices (ICH-GCP). All participants signed informed consents upon enrollment. The study was prospectively registered at clinicaltrials.gov prior to enrollment (NCT05498662). The findings reported are based on data from a single center participating in an ongoing multi-center study. The manuscript adheres to the STROBE guidelines [20]. All patients received a dental implant (T3^®^ PRO, ZimVie, Palm Beach Gardens, FL, USA) that has progressively increased thread depth with a contemporary hybrid surface topography. Patients were screened for inclusion in the study, which included collecting medical and dental histories and obtaining a CBCT scan. The following inclusion criteria were used:

1. Patients at least 18 years of age requiring 1–2 single dental implants in edentulous sites (only non-incisor sites).
2. Sites were considered for immediate extraction and implant placement if the sites were classified as Type I extraction sockets.
3. Previously healed edentulous ridges
4. For all sites, presence of opposing dentition that allows for functional occlusion.
5. At least 10 mm of alveolar bone height.

Patients were excluded if they met any of the following criteria:

1. Had uncontrolled systemic diseases, a history of bisphosphonate use, radiation to the head and jaw, HIV infection, or active hepatitis.
2. Had active infection or severe inflammation in the areas intended for treatment.
3. Were active smokers.
4. Had evidence of untreated severe parafunctional habits such as bruxing or clenching.
5. Had a history of untreated periodontitis.

### 2.1. Surgical Protocol

All surgeries were performed between August 2022 and November 2023 in the Post Graduate Periodontics Department at LSU by calibrated third-year residents under faculty supervision. Patients were pre-medicated with 2 g amoxicillin (or 600 mg clindamycin in case of allergy) 1 h prior to the start of the procedure. The implants were placed after reflection of a full-thickness flap and in accordance with the drilling protocols of the manufacturer in a prosthetically driven implant position. The insertion torque and ISQ were measured after placement of the implant. An immediate temporary restoration was placed on the day of implant placement if the implant reached primary stability (insertion torque > 35 N·cm and ISQ > 70) and the site was conducive for an immediate temporary as assessed clinically. These temporary prostheses were fabricated using custom-printed temporary shells made out of polymethyl methacrylate that were picked up intra-orally and delivered on the same day as the implant placement (a representative case is shown in Figure 1). They were designed to support an immediate loading protocol. All patients not qualified for immediate loading received a scannable healing abutment (BellaTek^®^ Encode^®^ Emergence; a representative case is shown in Figure 2). If an implant had an insertion torque of <20 N·cm, a cover screw was placed and left submerged for approximately 4 months. Patients received additional bone or soft tissue grafting to improve contours where indicated. The flap was sutured, and standardized radiographs were taken using protocols described below. Patients were followed up at 14 days and 28 days for assessment of healing.

After a healing period of approximately 3–4 months, all patients underwent digital impressions for fabrication of a permanent prosthesis. Approximately four to seven months after implant placement, all patients received a full-contour zirconia restoration that was fabricated either on a titanium base or a custom milled titanium abutment. On the day of prosthesis delivery, ISQ values, clinical measurements (probing depth, bleeding on probing, suppuration, implant mobility, peri-implant radiolucency, plaque index, gingival index), and a standardized radiograph were obtained. Additionally, a patient satisfaction questionnaire (addressing satisfaction with overall treatment, eating, speaking, and fit of the prosthesis, and overall comfort with the new prosthesis) was administered. Patients then returned at 1 and 2 years after implant placement, and clinical measurements (probing depth, bleeding on probing, suppuration, implant mobility, plaque index, gingival index) and standardized radiographs were assessed again. The implant survival and success rates were computed at the 1- and 2-year follow-up visits. The success rate was assessed using Albrektsson’s criteria [21,22]. Prosthetic complications were assessed during the entire study period.

### 2.2. Radiographic Analysis

For the measurement of crestal bone remodeling, standardized digital radiographs were included in the analyses. All radiographs were taken with a parallel cone technique using a custom-made stent for every patient fabricated after implant placement and retained for the duration of the study. Crestal bone landmarks on each radiographic image were marked by a trained and qualified clinician evaluator who was blinded to the study design. On both the mesial and distal aspects of the implant, the evaluator scored two marks designating where the crestal bone intersected the implant body apically and coronally. A reference landmark across the implant–abutment interface was identified, and the marginal bone level measurements were made using software (OsiriX MD version 14, Pixmeo SARL, Bernex, Switzerland). An average of the mesial and distal measurements was used for calculating bone-level changes for the implant over time. A decrease in this distance represents a coronal shift in marginal bone level, whereas an increase represents an apical shift. A calibration step was performed using known implant dimensions (implant length or collar width) and the observed implant dimensions to normalize and compensate for possible angulation and proximity effects. The marginal bone levels were measured at implant placement, permanent prosthesis delivery, and the 1-year and 2-year visits.

### 2.3. Patient-Reported Outcomes

A study-specific patient satisfaction questionnaire was used to assess patient-reported outcomes related to implant-supported prosthetic rehabilitation (full questionnaire available in the Supplementary Materials). The questionnaire evaluated overall satisfaction, comfort, satisfaction with eating and speaking, ability to taste food, and satisfaction with prosthesis stability. Responses were recorded using a three-point Likert scale (1 = excellent, 2 = good, 3 = poor). The questionnaire was administered at prosthesis delivery and at the 1- and 2-year follow-up visits. Overall satisfaction, satisfaction when speaking, and satisfaction with prosthesis stability were evaluated at all assessment time points. Comfort during chewing, satisfaction when eating, and ability to taste food were obtained at the annual follow-up visits and not assessed at prosthesis delivery. The questionnaire domains were informed by constructs commonly assessed in oral health-related quality-of-life and patient satisfaction instruments, including those represented in the Oral Health Impact Profile (OHIP-14) and relevant to implant therapy. However, the questionnaire itself was not independently validated and was intended solely to provide a descriptive assessment of patient satisfaction rather than a validated measure of oral health-related quality of life.

### 2.4. Statistical Analysis

Continuous variables were summarized as means ± standard deviations (SDs), with additional reporting of medians and ranges. Categorical variables were summarized as frequencies and percentages. Changes in peri-implant bone levels between prosthesis loading and 1-year or 2-year follow-up as well as between 1-year and 2-year follow-up were evaluated using paired t-tests. Cases with missing data at specific time points were excluded pairwise from the corresponding analyses. Statistical significance was set at a two-sided alpha level of 0.05. One participant received two implants. Primary analyses were performed at the implant level, treating implants as independent observations. A sensitivity analysis excluding one implant from this participant (who contributed two implants) yielded similar results and did not change study conclusions.

To assess factors associated with study outcomes, exploratory univariate analyses were performed. Primary outcomes were changes in radiographic bone levels from prosthesis delivery to 1 year, from prosthesis delivery to 2 years, and changes from 1 and 2 years. Secondary outcomes included implant survival and success, implant stability, peri-implant clinical parameters, prosthetic complications, and patient-reported satisfaction. Secondary exploratory analyses were performed to identify variables associated with insertion torque, ISQ, and marginal bone-level changes. To identify factors associated with these outcomes, exploratory univariate analyses were performed for each predictor variable, including age, gender, average ISQ, insertion torque, and anatomical location. Prosthesis type and soft tissue grafting were additionally evaluated as predictors of bone level changes from prosthesis delivery to 1 year and 2 years. Univariate analysis was conducted with simple linear regression models (using the lm( ) function in R). Each predictor was analyzed separately to examine its association with the continuous outcome variable. A formal multivariate analysis was not performed due to the limited sample size and exploratory nature of the predictive analysis. All statistical analyses were performed using R (4.5.2).

## 3. Results

A total of 30 patients receiving 31 implants (one patient received two implants) were recruited for the study. All patients completed all study visits except one patient who missed the 2-year follow-up visit for unknown reasons. The average age of the participants was 60 ± 12 years of age, and 14 male and 16 female participants were included. A total of 19 implants were placed in the maxilla and 12 in the mandible. Three of those sites (two maxillary, one mandibular) received immediate implant placement with extraction. Immediate loading was performed in eight sites (26%). A single stage procedure with conventional loading was done in 17 sites (54.8%), and a two-stage procedure with conventional loading occurred in six sites (19%). The average insertion torque was 39.96 ± 20.30 with 58% of implants reaching >35 N·cm insertion torque values. The average implant stability quotient (ISQ) measured at implant placement was 72 ± 14 (Table 1). When comparing immediate loaded implants versus delayed loaded implants, the average insertion torque was 57 ± 20 N·cm versus 34 ± 17 N·cm, respectively, and average ISQ was 82 ± 5 versus 68 ± 14, respectively. Bone grafting covered with an allograft membrane was performed at 11 of 31 implant sites (35.5%), while soft tissue grafting was performed at 12 of 31 implant sites (38.7%). The average time from implant placement to impression was 4.3 months ± 1.6, and that from implant placement to final prosthesis delivery was 5.4 months ± 1.8. Among the 19 maxillary implants, 10 (52.6%) were restored using cement-retained prostheses, and nine (47.4%) were restored using screw-retained prostheses. Among the 12 mandibular implants, nine (75.0%) were restored using screw-retained prostheses, and three (25.0%) were restored using cement-retained prostheses. ISQ values measured at prosthesis delivery averaged 81 ± 4. High levels of patient satisfaction with the prosthesis (mean scores ranging from 1.03 to 1.13) were observed at all time points (prosthesis delivery and 1-year and 2-year follow-ups) (Table 2, Supplemental Table 1).

Mean peri-implant probing depth remained stable during follow-up, measuring 2.91 ± 0.91 mm at 1 year and 2.86 ± 1.21 mm at 2 years. Minimal changes were observed in the probing depths between 1 and 2 years (mean difference: −0.06 ± 1.06 mm). At 1 year, BOP was detected at 41.9% (13/31) of implants, with mean gingival index and plaque index scores of 0.32 ± 0.48 and 0.65 ± 0.66, respectively. At 2 years, BOP was present at 46.7% (14/30) of implants. Mean GI and PI scores at the 2-year evaluation were 0.63 ± 0.67 and 0.57 ± 0.63, respectively. No implants demonstrated suppuration at either the 1-year or 2-year follow-up evaluations. Likewise, no implant mobility or peri-implant radiolucency was observed during all time points assessed.

### 3.1. Implant and Prosthesis Survival

All patients attended the follow-up visits, except one patient who failed to attend the 2-year follow-up and was excluded from the 2-year results. All implants survived the 2-year follow-up, and the observed implant survival rate was 100% (30/30; one patient lost to follow-up). Prosthetic complications were observed in 16% of implants (n = 5/31) with the most common being crown loosening (n = 2) and decementation (n = 2) and zirconia superstructure fracture (n = 1). All complications were handled without additional damage to the implant surface or abutment. A total of six other soft tissue complications (n = 6/31) were reported. The soft tissue complication was infection (n = 3), early cover screw exposure (n = 1), and soft tissue sensitivity (n = 2). All soft tissue complications were handled without any adverse reaction. The implant success rate at 2-year follow-up was found to be 96.67% (29/30; one patient had annual bone loss progression > 0.2 mm) (Table 3).

### 3.2. Radiographic Marginal Bone Levels

Mean marginal bone levels remained stable at the 1-year follow-up, with the mean change from prosthesis delivery to 1 year being negligible (+0.00 ± 0.26 mm; *p* = 0.994). Between prosthesis delivery and the 2-year follow-up, the mean change was −0.168 mm (95% CI: −0.26 to −0.07 mm; *p* = 0.001). Between the 1- and 2-year follow-up visits, the mean change was −0.17 ± 0.15 mm (*p* < 0.0001), indicating a small coronal shift in radiographic marginal bone level during the second year of follow-up (Table 3).

From prosthesis delivery to the 1-year follow-up, univariate analysis showed that a higher average ISQ was significantly associated with smaller bone-level changes (β = −0.009, 95% CI: −0.015 to −0.003; *p* = 0.004), but the magnitude of the association was small.

From prosthesis delivery to the 2-year follow-up, univariate analyses again demonstrated a small negative association with ISQ (β = −0.009, 95% CI: −0.0150 to −0.0034; *p* = 0.003).

For insertion torque, univariate analyses showed that average ISQ (β = 0.603, 95% CI: 0.09 to 1.11, *p* = 0.022) was positively associated with higher final torque. For buccolingual ISQ (ISQ1), bone grafting was associated with lower values (β = −12.400, CI: −22.34 to −2.46, *p* = 0.016). For mesiodistal ISQ (ISQ2), univariate analyses showed that final torque (β = 0.337, 95% CI: 0.077 to 0.60, *p* = 0.013) was positively associated, whereas bone grafting was negatively associated (β = −15.000, CI: −26.06 to −3.94, *p* = 0.010).

## 4. Discussion

This prospective cohort study evaluated 2-year clinical, radiographic, and patient-reported outcomes following placement of a tapered implant system with a contemporary hybrid surface. High implant survival and success rates were observed (100% and 96.67%, respectively), with stable marginal bone levels and favorable patient-reported outcomes. No implant failures were observed across different loading protocols during the 2-year observation period; however, the study was not designed or powered to compare loading protocols.

The observed survival rates are consistent with those reported for implant systems, where survival commonly exceeds 90–95% [7,23,24]. Peri-implant probing depths remained stable over time. The incidence of biological complications observed in this cohort is consistent with short-term studies, but longer follow-up is required to assess the development of peri-implant disease. The prosthetic complications observed align with previous reports indicating that technical complications—particularly loss of retention and component loosening—remain relatively common in implant-supported restorations [25,26,27,28].

The outcomes observed in the present study are broadly consistent with reports evaluating modern tapered implant systems featuring aggressive thread geometries. Aggressively threaded implant designs have been shown to improve insertion torque that allows for confidence in achieving primary implant stability while facilitating immediate or early loading protocols. Specifically, one study evaluating such a tapered aggressively threaded implant design reported a mean insertion torque of 39 N·cm [29] that was comparable to the insertion torque values obtained in this study despite the different microgeometry of the implant used in the two studies. Another study reported a higher insertion torque of 58 N·cm and good performance in both immediate and delayed placement protocols [30]. A recent systematic review [31] concluded that increased thread depth and tapered geometry are associated with improved primary stability and high survival rates, while marginal bone loss remains within clinically acceptable limits when appropriate protocols are followed. These findings are consistent with the present study, where high survival rates and stable marginal bone levels were observed.

Radiographic analysis demonstrated minimal marginal bone level changes following prosthesis delivery. This pattern is consistent with established evidence indicating that the majority of peri-implant bone remodeling occurs during early healing, with limited changes thereafter [32,33,34]. The small change observed between 1 and 2 years, although statistically significant, was small in magnitude. Primary stability, assessed using insertion torque and ISQ, played an important role in treatment planning. Implant stability has been widely recognized as an important determinant of osseointegration and determine loading protocols [9,32]. As expected from the prespecified treatment criteria, implants selected for immediate loading demonstrated higher insertion torque and ISQ values. These differences reflect the clinical selection protocol and should not be interpreted as an effect of loading strategy on primary stability. Existing evidence indicates that implant macrogeometry may enhance primary stability, particularly in low-density bone, although its direct influence on marginal bone outcomes remains difficult to isolate [31]. No implant failures were observed among the eight immediately loaded implants during the 2-year observation period; however, the small, non-randomly selected subgroup precludes comparative conclusions regarding loading protocols. This observation is consistent with randomized trials and systematic reviews demonstrating similar survival rates and marginal bone levels between immediate and delayed loading protocols [35,36]. However, interpretation is limited by the relatively small number of immediate loading cases and inherent selection bias.

Lower ISQ values in grafted sites likely reflect underlying differences in bone quality and ridge morphology. Previous studies have demonstrated that implant stability is influenced by bone density and local site characteristics [32,37]. Patient-reported outcomes indicated high levels of satisfaction across all evaluated domains, including comfort, function, and prosthesis stability. These findings reinforce the clinical success of the implant therapy from the patient perspective and highlight the importance of incorporating patient-centered outcomes into implant research.

Prosthetic complications occurred in five of 31 restorations (16.1%) and consisted primarily of crown loosening and decementation. Although these events did not result in implant loss or compromise implant success, they represent clinically relevant maintenance requirements that warrant consideration when evaluating overall treatment outcomes. These events represented manageable technical complications and were resolved without compromise of implant success. Previous studies and reviews have shown that screw loosening and loss of retention remain among the most frequently reported technical complications associated with implant-supported restorations, even when implant survival remains high [38,39].

This study has several strengths, including its prospective design, standardized surgical and restorative protocols, and comprehensive assessment of clinical, radiographic, and patient-reported outcomes. The use of calibrated radiographic measurements and blinded evaluators further enhances the reliability of the findings. However, a formal reliability assessment for radiographic measurements was not conducted, and this represents a limitation. The inclusion of clinician-selected loading and grafting protocols reflects variation encountered in clinical implant treatment. A principal limitation of this study is the absence of a comparator group. Consequently, the findings should be interpreted as descriptive performance outcomes for a single-implant system rather than evidence supporting superiority or equivalence relative to other implant designs, surfaces, or treatment protocols. Although favorable clinical, radiographic, prosthetic, and patient-reported outcomes were observed, the present study cannot determine whether these outcomes were attributable to specific implant design characteristics or whether similar results would have been achieved with alternative implant systems. Comparative randomized or controlled prospective studies are needed to evaluate the relative contribution of implant design features to clinical outcomes. Other limitations include the single-center design and relatively small sample size, which limit the statistical power of subgroup analyses and the generalizability of the findings. The number of immediate placement and immediate loading cases was limited, restricting definitive comparisons. Furthermore, the modest sample size precluded robust multivariable modeling and limited the ability to perform meaningful subgroup comparisons. Finally, the 2-year follow-up period may not capture long-term complications or the incidence of peri-implantitis. Although the sponsor had no role in study conduct, analysis, interpretation, manuscript preparation, or publication decisions, the findings should be interpreted within the context of a manufacturer-supported investigation and warrant confirmation in larger independently funded studies.

The results of this study suggest that the investigated implant system demonstrated favorable short-term outcomes across the clinical scenarios represented in this cohort. The findings also emphasize the importance of objective stability measures, such as insertion torque and ISQ, in guiding loading decisions. Future research should include larger, multicenter studies with extended follow-up periods to confirm these findings and assess longer-term implant performance. Further investigations are also warranted to evaluate the biomechanical impact of progressive thread designs.

Within the limitations of this prospective single-center pilot cohort, the contemporary hybrid surface tapered implant system demonstrated acceptable clinical performance over 2 years, with 100% observed implant survival, limited marginal bone-level changes, and acceptable patient satisfaction.

## Figures and Tables

**Figure 1:**
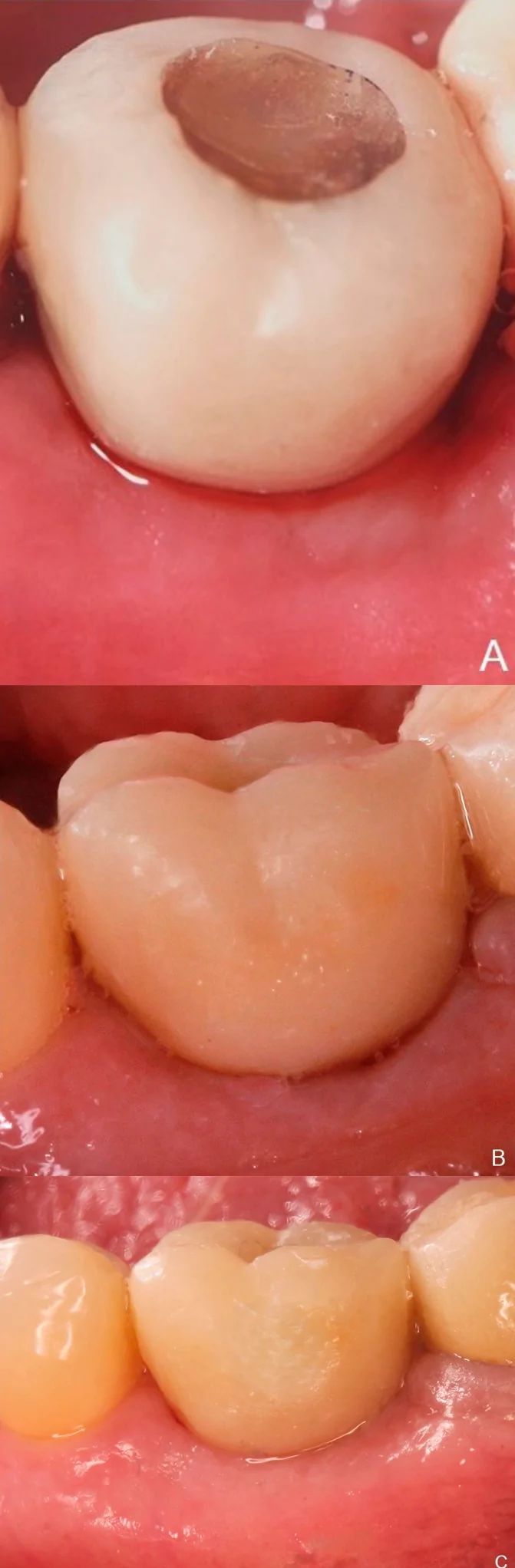

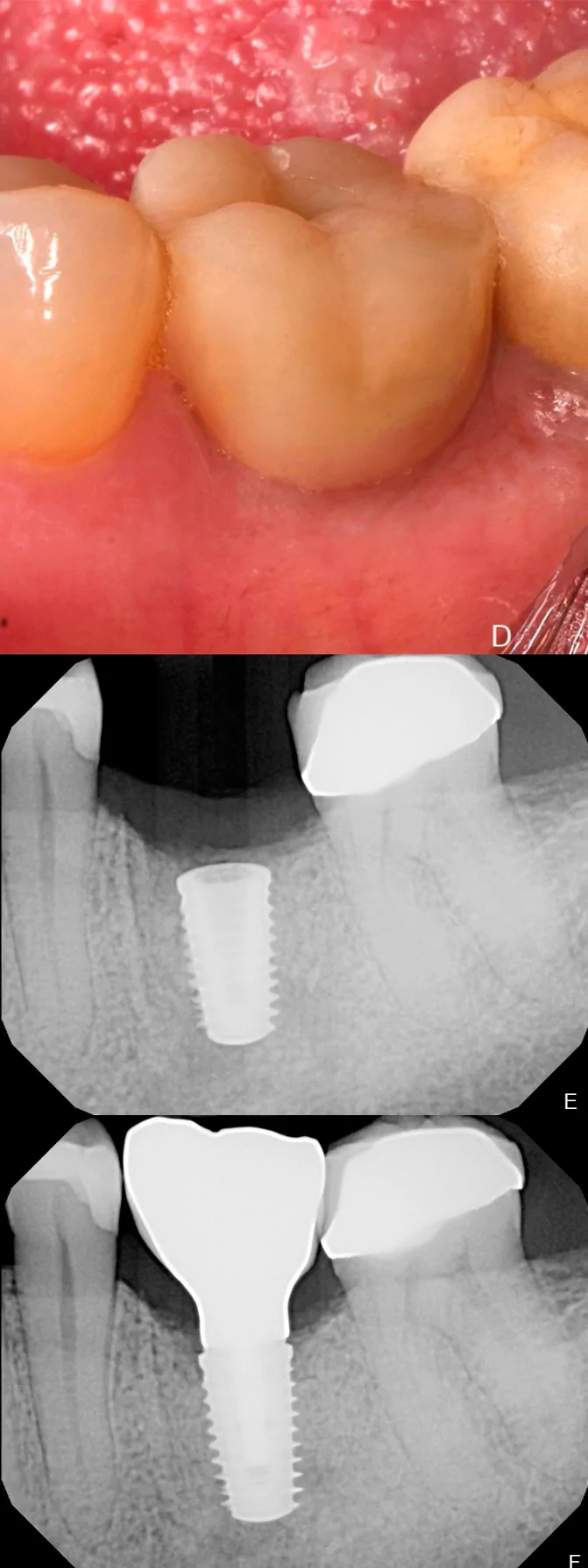

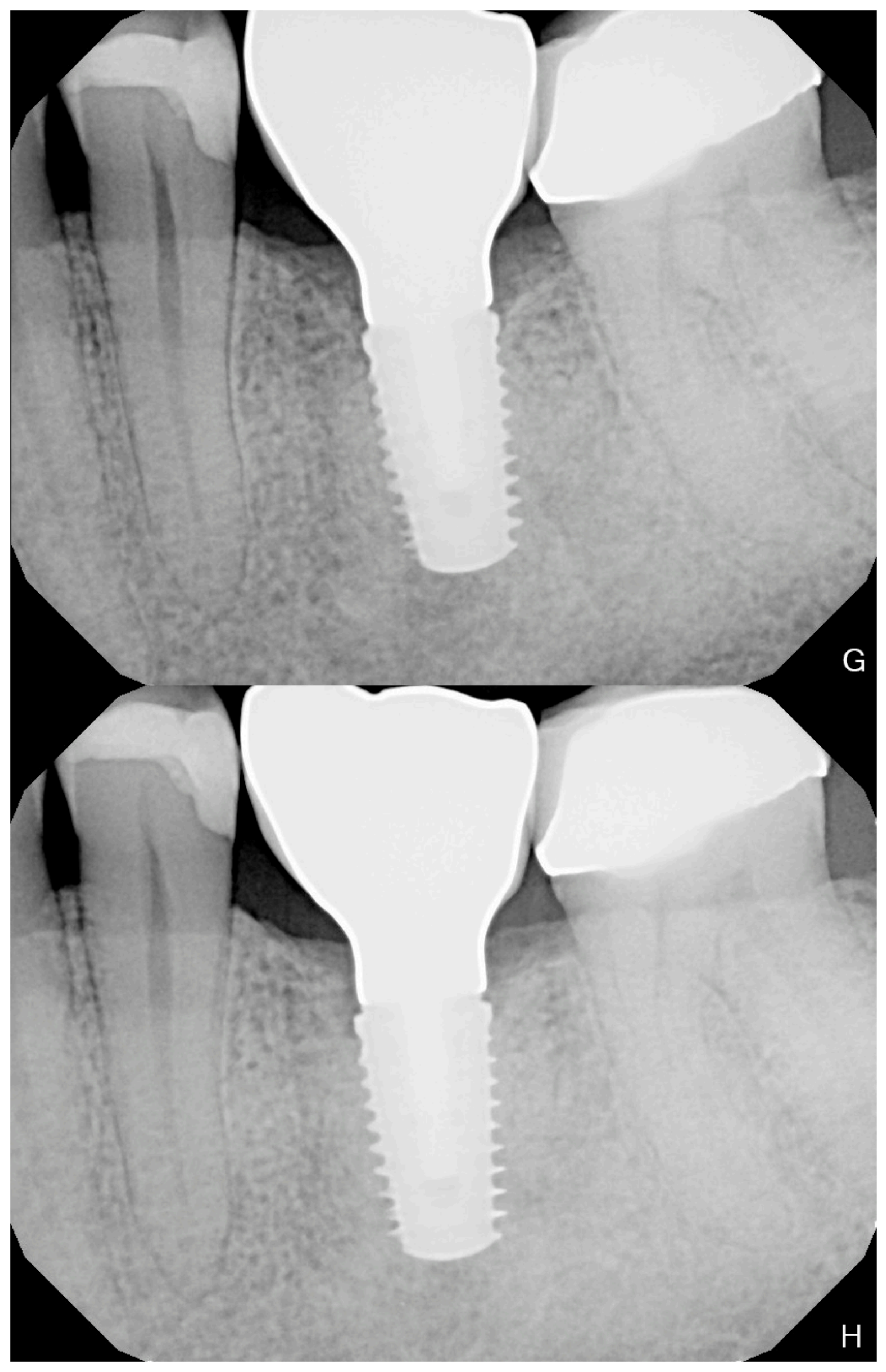
A representative series of a patient receiving immediate loading protocol. (**A**) Image represents the immediate temporary in place. A final crown was fabricated 4 months later (**B**). Follow-up was carried out at 1 year (**C**) and 2 years (**D**). Serial radiographs at implant placement (**E**), crown delivery (**F**), 1-year follow-up (**G**), and 2-year follow-up (**H**) show stable bone levels.

**Figure 2:**
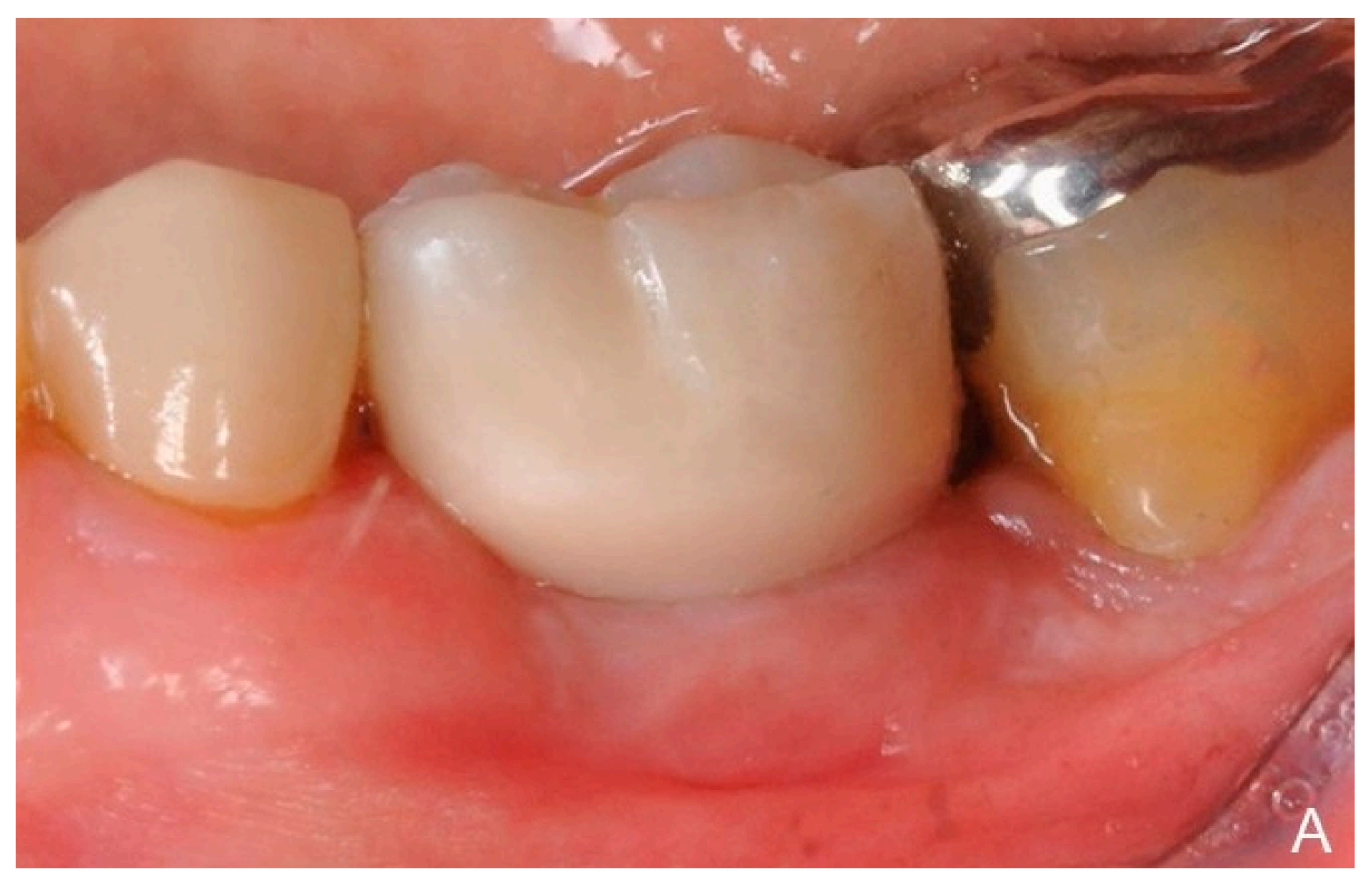

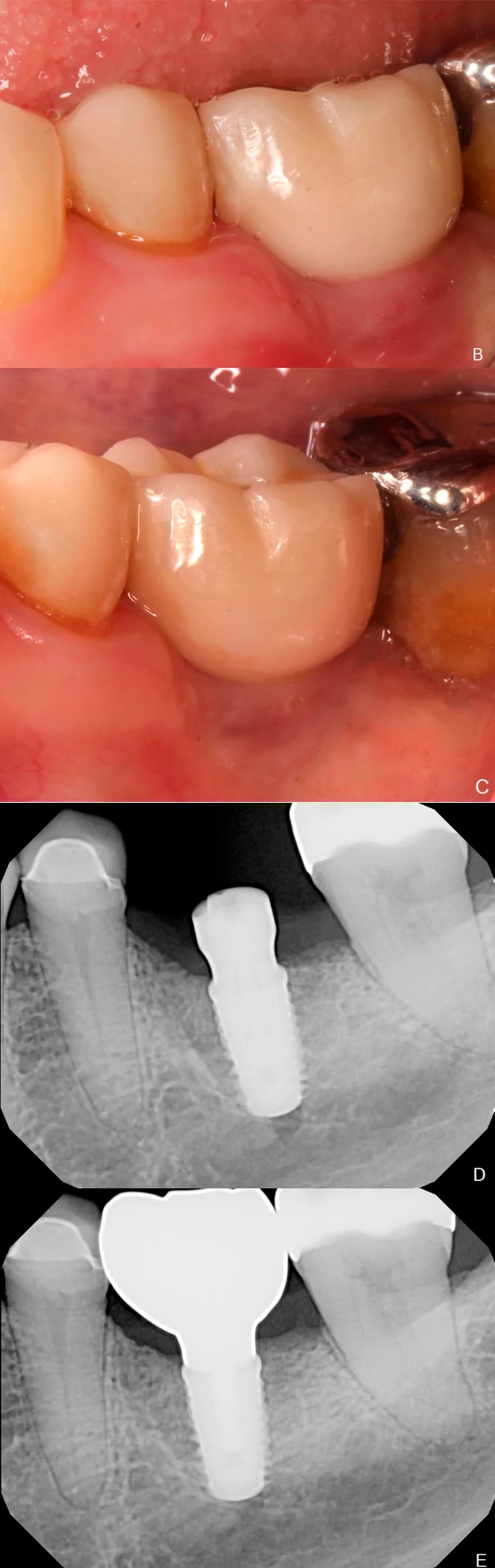

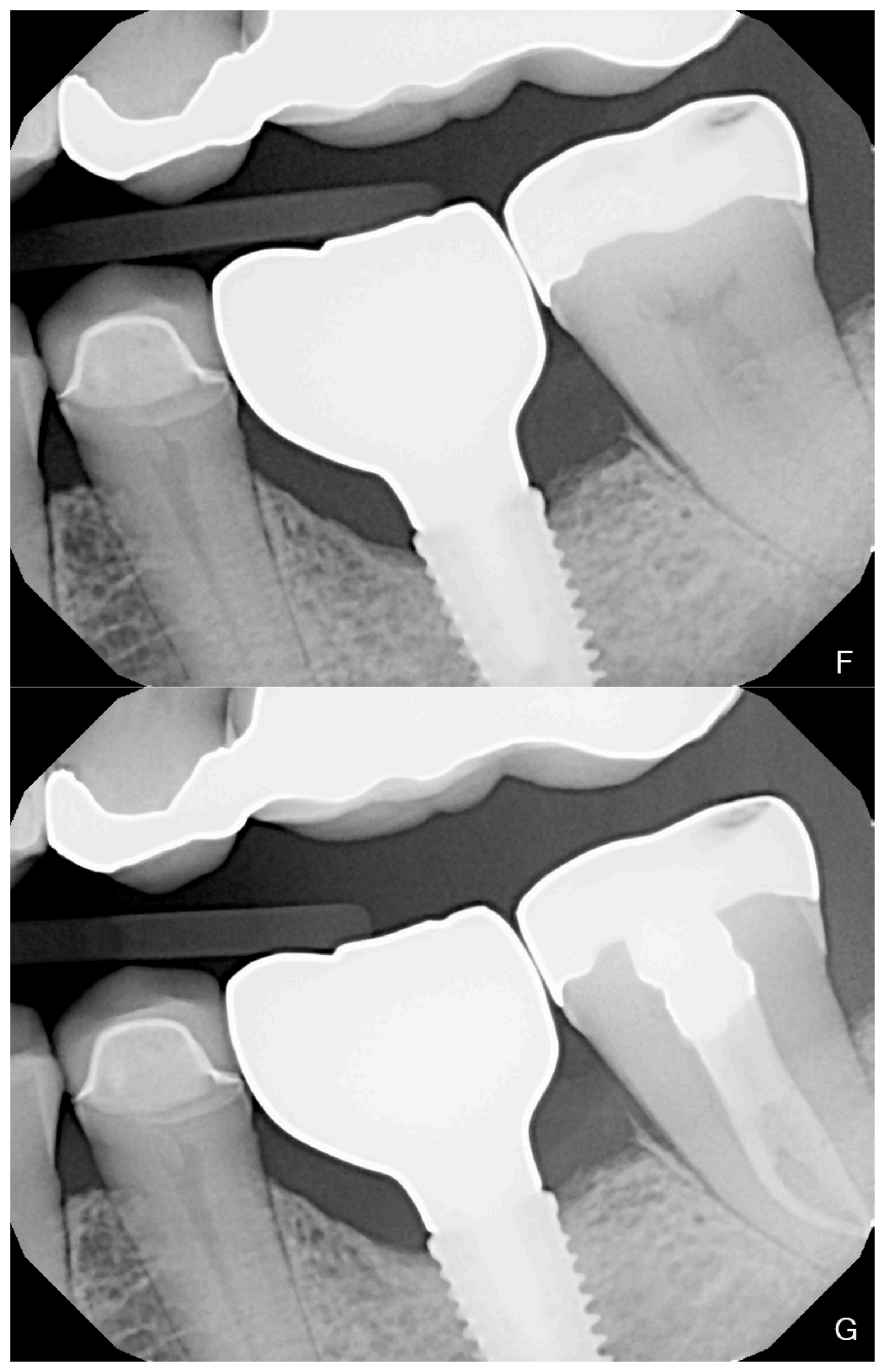
A representative series of a patient receiving delayed loading protocol. (**A**) Image represents the final crown followed by 1-year (**B**) and 2-year post-op (**C**). Serial radiographs at implant placement (**D**), crown delivery (**E**), 1-year follow-up (**F**) and 2-year follow-up (**G**) show stable bone levels.

**Table 1:** Patient and site characteristics.

Variable	Value
Number of patients	N = 30
Number of implants	N = 31
Mean age ± SD	60 ± 12
Sex (M/F)	14/16
Maxilla/mandible	19/12
Immediate loading	N = 8
Delayed loading	N = 23
Immediate placement	N = 3
Healed site placement	N = 28
Bone grafting	N = 11
Soft tissue grafting	N = 12
Mean insertion torque ± SD	39.96 ± 20.30
Mean ISQ1 ± SD	72.26 ± 13.45
Mean ISQ2 ± SD	71.13 ± 15.23

**Table 2:** Patient-reported outcomes.

Domain	Mean ± SD (Prosthesis Placement)	Mean ± SD (1 Year)	Mean ± SD (2 Year)
Overall satisfaction	1.03 ± 0.18	1.07 ± 0.25	1.07 ± 0.26
Comfort (pain on chewing)	N/A *	1.10 ± 0.31	1.10 ± 0.31
Satisfaction when eating	N/A *	1.13 ± 0.35	1.10 ± 0.31
Satisfaction when speaking	1.03 ± 0.18	1.03 ± 0.18	1.07 ± 0.26
Ability to taste food	N/A *	1.07 ± 0.25	1.07 ± 0.26
Satisfaction with stability (the fit in mouth)	1.1 ± 0.31	1.13 ± 0.35	1.07 ± 0.26

* Comfort during chewing, satisfaction when eating, and ability to taste food were not assessed at prosthesis delivery and were first evaluated at the 1-year follow-up.

**Table 3:** Clinical and radiographic outcomes.

Outcome	Value
Implant survival (% at 2 years)	100% (30/30)
Prosthetic complications (%)	16%
Implant success (% at 2 years)	96.67% (29/30)
Mean PD at 1 year ± SD	2.90 ± 0.91
Mean PD at 2 years ± SD	2.86 ± 1.21
Mean bone change restoration → 1 year ± SD	0 ± 0.26
Mean bone change restoration → 2 years ± SD	−0.17 ± 0.25

## Data Availability

All data supporting the conclusions of this study are available from the corresponding author upon reasonable request.

## Supplementary Materials

The following supporting information can be downloaded at: https://www.mdpi.com/article/10.3390/jcm15218691/s1, Figure S1: Patient reported outcomes questionnaire; Table S1: Patient reported outcomes presented as % of high scoring respondents.

## Abbreviations

The following abbreviations are used in this manuscript:

**Abbreviation**	**Definition**
CBCT	Cone-Beam Computed Tomography
DAE	Dual Acid-Etched
HIV	Human Immunodeficiency Virus
ICH-GCP	International Conference on Harmonization—Good Clinical Practice
IRB	Institutional Review Board
ISQ	Implant Stability Quotient
OHIP-14	Oral Health Impact Profile-14
PD	Probing Depth
SD	Standard Deviation
STROBE	Strengthening the Reporting of Observational Studies in Epidemiology
