# Supplementary material for: Clinical Performance of a Contemporary Hybrid Surface Implant System—A Prospective Pilot Study
Source: J Clin Med. 2026 Nov 7;15(21):8691. doi: 10.3390/jcm15218691 (PMC13764123; doi:10.3390/jcm15218691)
Supplement: Supplementary file 1 [file jcm-15-08691-s001.zip › jcm-4628809-supplementary.pdf]

Figure S1. Patient reported outcomes questionnaire

	Excellent	Good	Poor
Patient's satisfaction with the overall treatment (new prosthesis/ teeth)	☐	☐	☐
Patient's comfort (any pain when chewing) with their new Prosthesis (teeth)	☐	☐	☐
Patient's satisfaction when eating with their new prosthesis (teeth)	☐	☐	☐
Patient's satisfaction when speaking with their new prosthesis (teeth)	☐	☐	☐
Patient's ability to taste food with their new prosthesis (teeth)	☐	☐	☐
Patient's satisfaction with the stability (fits secure in the mouth) of their new prosthesis (teeth)	☐	☐	☐

Supplemental Table S1: Patient reported outcomes presented as % of high scoring respondents

Domain	Prosthesis Delivery (n=30) Score 1	Score 2	1 Year (n=30) Score 1	Score 2	2 Years (n=29)* Score 1	Score 2
Overall satisfaction	29 (96.7%)	1 (3.3%)	28 (93.3%)	2 (6.7%)	27 (93.1%)	2 (6.9%)
Comfort	Not collected	Not collected	27 (90%)	3 (10%)	26 (89.7%)	3 (10.3%)
Satisfaction when eating	Not collected	Not collected	26 (86.7%)	4 (13.3%)	26 (89.7%)	3 (10.3%)
Satisfaction when speaking	29 (96.7%)	1 (3.3%)	29 (96.7%)	1 (3.3%)	27 (93.1%)	2 (6.9%)

Ability to taste food	Not collected	Not collected	29 (96.7%)	1 (3.3%)	27 (93.1%)	2 (6.9%)
Satisfaction with stability (fit in mouth)	27 (90%)	3 (10%)	26 (86.7%)	4 (13.3%)	26 (93%)†	2 (7%)†

*One subject was lost to follow-up at 2 years.

† One subject had a missing response for the stability question at 2 years, resulting in an available denominator of 28 rather than 29.
